# Efficiency of the Stool-PCR Test Targeting NADH Dehydrogenase (*Nad*) Subunits for Detection of *Opisthorchis viverrini* Eggs

**DOI:** 10.1155/2021/3957545

**Published:** 2021-12-06

**Authors:** Wansika Phadungsil, Supaporn Pumpa, Kridsada Sirisabhabhorn, Amornrat Geadkaew-Krenc, Rudi Grams, Mathirut Mungthin, Toon Ruang-Areerate, Poom Adisakwattana, Nipawan Labbunruang, Pongsakorn Martviset

**Affiliations:** ^1^Graduate Program in Biomedical Sciences, Faculty of Allied Health Sciences, Thammasat University, Pathumthani 12120, Thailand; ^2^Department of Medical Technology Laboratory, Thammasat University Hospital, Pathumthani 12120, Thailand; ^3^Department of Phamacology, Phramongkutklao College of Medicine, Bangkok 10400, Thailand; ^4^Department of Parasitology, Phramongkutklao College of Medicine, Bangkok 10400, Thailand; ^5^Department of Helminthology, Faculty of Tropical Medicine, Mahidol University, Bangkok 10400, Thailand; ^6^Department of Biology, Faculty of Science, Udon Thani Rajabhat University, Udon Thani 41000, Thailand; ^7^Division of Parasitology, Department of Preclinical Science, Faculty of Medicine, Thammasat University, Pathumthani 12120, Thailand

## Abstract

*Opisthorchis viverrini* infection is the major parasitic infection problem in Southeast Asian countries, and long-term infection will lead to cholangiocarcinoma (CCA), the bile duct cancer. The early diagnosis of *O*. *viverrini* infection may interrupt the progression of the opisthorchiasis and other related illnesses, especially CCA. The current diagnostic procedure is stool examination by microscope-based methods such as direct smear and concentration techniques but it is limited by low parasite egg numbers. The molecular diagnosis prompts the chance to evaluate the light infection with low number of parasite eggs but is currently inconvenient for routine use due to special equipment requirement and unstable sensitivities. Our present study aims to establish the efficiency of *OvNad* subunits, the mitochondrial gene, for introducing as a potential diagnostic target by conventional PCR, the cheapest and easiest molecular procedure. A total of 166 stool samples were investigated microscopically by the PBS-ethyl acetate concentration technique (PECT); 75 samples were *O*. *viverrini* positive with 28 samples that were positive with single parasite (hookworm, *A*. *lumbricoides*, *S*. *stercoralis*, *Taenia* spp., and *T*. *trichiura*), 11 samples were with mixed infection, and 52 samples were without parasite detection. The detection limits of *OvNad* subunits were evaluated in artificially spiked samples containing 0, 1, 5, 10, 20, 50, and 100 *Ov*-eggs. The result suggested that the best detection efficacy was of *OvNad5* that had exact detection limits at only 5 eggs. In the PCR amplification of *OvNad* subunits, there exist 100% specificities with varied sensitivities from 64%, 88%, 80%, and 100% of *OvNad1*, *OvNad2*, *OvNad4*, and *OvNad5*, respectively. *OvNad* subunits were amplified specifically without cross reactivity with the other collected parasites. Our study established that *OvNad* subunits, especially *OvNad5*, are the potent candidates for PCR amplification of stool containing *Ov*-eggs with high confidential sensitivity, specificity, PPV, and NPV even in the light infection that would be a benefit for developing as a routine diagnosis of *O*. *viverrini* infection.

## 1. Introduction

*Opisthorchis viverrini (O*. *viverrini*; *Ov)*, a digenean liver fluke, is a major cause of human opisthorchiasis and *Ov*-induced cholangiocarcinoma (CCA), the biliary ductal cancer, in enduring infection [1]. It is a food-borne parasite that spreads throughout the Southeast Asian subregional countries, especially in the Mekong Basin including Laos PDR, Cambodia, Vietnam, and Thailand [2–4]. Huge incidence of *O*. *viverrini* is highly related to the prevalence of CCA in those areas according to the consumption of raw Cyprinid fish, a secondary intermediate host of *O*. *viverrini* [5]. CCA is a highly progressive cancer originating from the transformed intra- or extrahepatic cholangiocytes which lack early diagnostic markers [6]. The diagnosis of *O*. *viverrini* infection is possible, but it has a lot of limitations. The gold standard for diagnosis of *O*. *viverrini* infection is microscope-based methods such as direct smear and concentration techniques including the formalin-ethyl acetate concentration technique (FECT) and related procedures [7]. The conventional methods are convenient but have low sensitivity; moreover, the observation could be erroneous and need professional laboratorians for identification by their naked eyes [8].

The molecular detection of *Ov*-eggs has been introduced for several years by many methods such as conventional PCR, LAMP, and quantitative real-time PCR [9–14]. The target genes are varied but mostly concentrated on the mitochondrial gene due to the presence of high conservation such as internal transcribed spacer (*ITS*), cytochrome c oxidase 1 (*cox1*), and NADH dehydrogenase 1 (*nad1*) [15–18]. The detection limit of those genes is not stable among the specimen types, collection procedures, preservation methods, extraction protocols, and the number of eggs [19].

**Figure 1:**
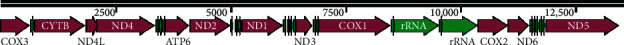
Schematic drawing of the *O*. *viverrini* mitochondrial genome showing the composition of *Nad* subunits (ND).

NADH dehydrogenase (Nad) is the enzyme which converts nicotinamide adenine dinucleotide (NAD) from NADH (reduced form) to NAD^+^ (oxidized form) encoded by the mitochondrial genome [20]. The NADH dehydrogenase complex spans inside the inner membrane of the mitochondria. The complex provides energy from the oxidation of NADH to NAD^+^ and proton transfer from the mitochondrial matrix to the intermembrane space. Moreover, the complex has numerous metabolic functions such as fatty acid oxidation, TCA cycle, amino acid catabolism, and cytosolic reactions given the crucial central position of metabolic pathways responsible for carbohydrate, fatty acid, and amino acid catabolism [21]. As it has various vital functions for the cells, *Nad* is abundantly expressed in every cell which is a good compelling target for molecular detection. The established mitochondrial genome of *O*. *viverrini* permits the possibility to encounter the *Nad* subunits for development as the diagnostic target other than *Nad1* that was previously tested [17]. The schematic drawing of the *O*. *viverrini* mitochondrial genome is illustrated in Figure 1. Hence, this study aimed to establish the new target for the molecular identification of *Ov*-egg directly in the stool specimen by comparison of the efficiency of *Nad* subunits using conventional PCR which is the easiest and cheapest molecular procedure for developing a potential diagnostic target in the future.

## 2. Materials and Methods

### 2.1. Study Areas and Stool Collection

A total of 166 stool samples were collected from several provinces in the north, northeast, and central Thailand where *O*. *viverrini* is endemic in Chiang Mai, Phrae, Phetchabun, Nakhon Phanom, Pathumthani, and Chachoengsao provinces during 2018 to 2020. The participants living in the risk areas of these provinces were included in this study. All participants were educated and consented for a noninvasive stool collection procedure. The collected samples were transferred to the laboratory and kept in 4–10°C until processed by the concentration method.

### 2.2. PBS-Ethyl Acetate Concentration Technique (PECT)

All samples were subjected to the PBS-ethyl acetate concentration technique (PECT) as previously described [15] with a few modifications. In brief, one gram of each stool specimen was mixed with 10 mL of PBS, pH 7.4, and filtered through three layers of gauze pad. 3 mL of ethyl acetate was added to the filtrate and mixed thoroughly by inverting. The tube was centrifuged at 2,000*×g*, for 10 min, and the upper phases were removed. The sediment was investigated under the light microscope at 100 and 400 magnifications.

### 2.3. Genomic DNA Isolation

The genomic DNA of parasitic eggs was isolated from PECT sediments of stool. Before extraction, the sediments were autoclaved at 121°C, for 5 min to open the operculum of parasite eggs. The genomic DNAs were then extracted by using the QIAamp^®^ PowerFecal^®^ DNA kit (QIAGEN, Germany) according to the manufacturer's instructions with the final filtrates containing 50 *µ*L of eluted DNAs.

### 2.4. Primer Design and PCR Amplification

*OvNad* subunit specific primers for PCR amplification were designed from a partial sequence of *O*. *viverrini* mitochondrial DNA available in GenBank (accession number JF739555.1). The primer sequences of *Ovnad1*, Ov*nad*2, Ov*nad*4, and Ov*nad*5 are presented in Table 1. The expected amplicons of *Ovnad1*, Ov*nad*2, Ov*nad*4, and Ov*nad*5 were 204 bp, 198 bp, 200 bp, and 199 bp, respectively. PCR amplifications were performed by using GoTaq® Colorless Master Mix (Promega, USA) containing 3 *µ*L of genomic DNA and 25 pmole of each forward and reverse primer in a thermal cycler (Mastercycler nexus Eppendorf flexlid, Germany). The amplification steps included initial denaturation at 95°C for 5 min, followed by 35 cycles of denaturation at 95°C for 1 min, annealing at 55°C for 1 min, extension at 72°C for 1 min, and one cycle of a final extension at 72°C for 10 min. The PCR products were size separated on 2% agarose gel containing ViSafe Red Gel Stain (Vivantis, USA) using 1X TBE buffer at 100 V for 2 h. The PCR products were confirmed for their conformation by DNA sequencing service (Macrogen, Republic of Korea).

### 2.5. Detection Limit Determination of OvNad Subunits

The detection limits of *OvNad* subunits were evaluated by using various numbers of *O*. *viverrini* eggs from adult parasites obtained from the livers of infected hamsters (the animal ethics was approved by the Thammasat Animal Care and Use Committee (TU-ACUC), no. 024/2559, following the care and use of laboratory animals' guideline (NRC 2011). The *O*. *viverrini* eggs of 0, 1, 5, 10, 20, 50, and 100 were artificially inoculated with 250 mg of *O*. *viverrini*-negative stools and processed for genomic DNA isolation. PCR amplifications were performed by using GoTaq® Colorless Master Mix (Promega, USA) containing 3 *µ*L of genomic DNA and 25 pmole of each forward and reverse primer in a thermal cycler (Mastercycler nexus Eppendorf flexlid, Germany). The amplification steps included initial denaturation at 95°C for 5 min, followed by 35 cycles of denaturation at 95°C for 1 min, annealing at 55°C for 1 min, extension at 72°C for 1 min, and one cycle of a final extension at 72°C for 10 min. The PCR products were size separated on 2% agarose gel containing ViSafe Red Gel Stain (Vivantis, USA) using 1X TBE buffer at 100 V for 2 h. The PCR products were confirmed for their correct sequences by DNA sequencing service (Macrogen, Republic of Korea).

### 2.6. Cross-Reactivity Evaluation

The cross reactivities of *OvNad* subunit specific primers were simultaneously investigated in the collected stools. The PECT sediments with other parasites were monitored for the cross reactivity. The samples included both single and mixed infections with either flatworms or roundworms.

### 2.7. Statistical Analysis

The sensitivity, specificity, positive predicted value (PPV), and negative predicted value (NVP) of *OvNad* subunit detection by PCR in the stool were calculated as previously described [22] comparing with PECT as a gold standard method.

## 3. Result

### 3.1. Parasite Detected by the Modified PBS-Ethyl Acetate Concentration Technique (PECT)

From 166 stool samples, the PECT result revealed the positive samples including *O*. *viverrini* (75/166), hookworm (11/166), *Trichuris trichiura* (6/166), *Strongyloides stercoralis* (5/166), *Taenia* spp. (3/166), and *Ascaris lumbricoides* (3/166). Moreover, the mixed infection has also been identified which comprised hookworm + *A*. *lumbricoides* (5/166), hookworm + *T*. *trichiura* (1/166), *A*. *lumbricoides* + *Enterobius vermicularis* (2/166), *A*. *lumbricoides* + *T*. *trichiura* (1/166), and *Taenia* spp. + *E*. *vermicularis* (2/166). The rest of the samples were negative for PECT (52/166). The result is shown in Table 2.

### 3.2. Detection Limits of OvNad Subunits

**Figure 2:**
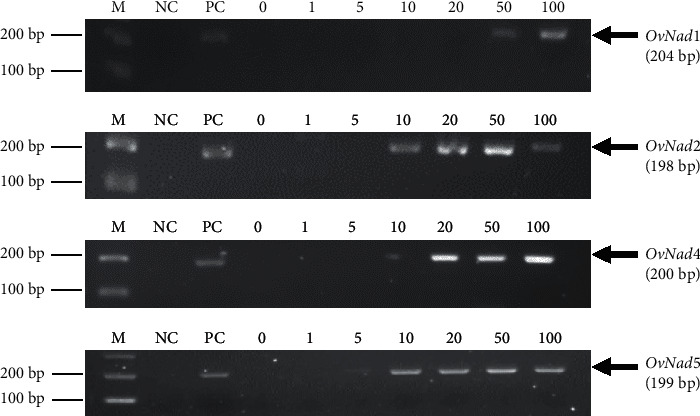
The detection limits of *OvNad* subunits in various numbers of eggs in artificially spiked samples (M, 100 bp DNA marker; NC, no template control; PC, positive control from uncountable *Ov*-eggs dissected from adult *O*. *viverrini*; and 0, 1, 5, 10, 20, 50, and 100 are the number of *Ov*-eggs inoculated in stool specimens).

The PCR amplification of *OvNad* subunits by conventional PCR with 0, 1, 5, 10, 20, 50, and 100 *Ov*-egg reviewed the different detection limits among the *OvNad* subunits. *OvNad1* has the lowest detection limit at 50 eggs followed by *OvNad4* at 20 eggs and *OvNad2* at 10 eggs. Interestingly, *OvNad5* had unyielding activities in the low egg number in only 5 eggs. The amplification of *OvNad* subunits is illustrated in Figure 2.

### 3.3. Sensitivity, Specificity, and Cross Reactivity of OvNad Subunits

**Figure 3:**
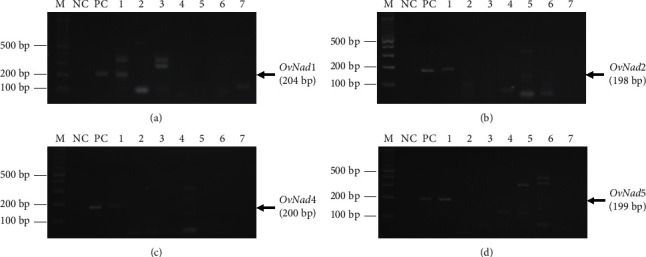
PCR amplicons of *OvNad* subunits using the new introduced primers and its cross-reactivity evaluation. (a) *OvNad1*, (b) *OvNad2*, (c) *OvNad4*, and (d) *OvNad5* (M, 100 bp plus DNA marker; NC, no template control, PC; positive control from uncountable *Ov*-eggs dissected from adult *O*. *viverrini*; and 1 to 7 are the samples processed by PECT, 1, *O*. *viverrini* egg detected sample; 2, parasite undetected sample; 3, hookworm egg detected sample; 4, *Ascaris lumbricoides* egg detected sample; 5, *Taenia* spp. egg detected sample; 6, *Strongyloides stercoralis* larva detected sample; and 7, *Trichuris trichiura* egg detected sample.

The PCR amplification of 166 samples with *OvNad* subunit specific primers suggested that *OvNad* subunits had different sensitivities. Only 48/75 of PECT-positive samples can be amplified by *OvNad1*, while 66/75 and 60/75 were successfully amplified by *OvNad2* and *OvNad4*, respectively. The *OvNad5* showed luminous activity with 75/75 of PECT-positive samples and another 2 from PECT-negative samples (the sequences had been correctly confirmed). The sensitivities of *OvNad1*, *OvNad2*, *OvNad4*, *and OvNad5* were 64.0%, 88.0%, 80.0%, and 100%, respectively, and specificities were 100% in all subunits. The positive predicted values (PPV) of *OvNad1*, *OvNad2*, *OvNad4*, and *OvNad5* were 100% in all subunits, whereas the negative predicted values (NVP) were 77.12%, 91.00%, 85.85%, and 100%, respectively. The sensitivities, specificities, PPV, and NPV are shown in Table 3. In the testing of cross reactivities, the *OvNad* subunits were used to amplify in all simultaneously collected parasites of both single and mixed infections. All *OvNad* subunits showed excellency without amplification of all other kinds of parasites as mentioned in Table 2 and Figure 3.

## 4. Discussion

The molecular technology allows many researchers to develop several diagnostic procedures for detecting of any diseases, especially infectious diseases with perilous complexity. The *O*. *viverrini* infection is also in the same pipeline with other diseases, which has some of established molecular diagnostic procedures, but the sensitivity has been limited. The ITS-based PCR is the first and most famously used target for molecular identification, but later, it showed varied sensitivity comparable with the newly identified targets such as *Cox1*, Cytochrome B (*CytB*), and *Nad1* [17]. Even though these targets showed better or equal sensitivities to ITS, they have many drawbacks, especially the detection in the low number of parasite eggs [10]. *Nad1* is a mitochondrial gene which has been previously introduced but never verified with the other *Nad* subunits. The detection limits of *OvNad* subunits were verified in our study. The result revealed that the *OvNad5* subunit had better activities than the other subunits. Only 5 eggs could be detected by *OvNad5* primer sets. When compared with the other established gene targets, also *OvNad* subunits, especially *OvNad5*, have better or equal capabilities to detect the lower concentration of eggs (5 EPG in artificially spiked samples) to *ITS-2* and *Cox1* [10, 11, 23, 24].

PECT, the stool concentration technique, is a routinely used method for investigating parasitic infection. It has shown the highest sensitivity than the other microscope-based methods up to 100% in >1000 eggs-per-gram (EPG) samples and exceeding 91% in cases of light infection [8]. As a result, we used PECT as a reference method for sensitivity, specificity, PPV, and NPV calculations. The best sensitivity from our study is of *OvNad5* which showed 100% sensitivity followed by *OvNad2*, *OvNad4*, and *OvNad1*, respectively. Surprisingly, the *OvNad2* and *OvNad5* revealed better sensitivities than *OvNad1* that was formerly established. *OvNad5* showed the best efficiency in all investigated parameters including sensitivity, specificity, PPV, and NPV with 100%. In comparison with the other target genes, *OvNad5* also has better capacity than *ITS-2* and *Cox1* mentioned in previous reports by Lamaningao [25] and Buathong [15], with 83.1% and 89.1%, respectively, especially in the light infection (less than 100 EPG) where the sensitivities of *ITS-2* and *Cox1* were up to 73.9% and 89.1% [10, 15, 23, 24]. DNA sequencing of the PCR products amplified by the introduced primers confirmed the sequences of *OvNad* that referred to the *Ov*-eggs with less identity to the minute intestinal fluke. The introduced stool PCR, especially *OvNad5*, showed a relevant result to natural *Ov*-positive samples investigated by PECT. All of the PECT-positive samples were also positive with *OvNad5* PCR; interestingly, two PECT-negative samples were positive with *OvNad5* PCR, emphasizing the limitation of the conventional method to detect the egg in low-intensity samples. For the cross reactivities to other parasites that could be found together with *Ov* infection including Hookworm, *A*. *lumbricoides*, *Taenia* spp., *S*. *stercoralis*, *E*. *vermicularis*, and *T*. *trichiura*, the result suggested that *OvNad* has no cross reactivity with these parasite eggs. It is confidentially confirmed that *OvNad* subunits could be the exact targets for PCR amplification of *Ov*-egg.

In conclusion, our study established that *OvNad* subunits can be the potential targets for *Ov*-egg DNA detection in the stool specimen. Moreover, *OvNad5* could be the best candidate due to the potent detection limit, highest sensitivity, specificity, PPV, and NPV. The introduced primers can be applied for using in routine diagnosis because conventional PCR is easier and cheaper than real-time PCR. It is also useful for other researchers to develop more tests on *OvNad* subunits in the future.

## Figures and Tables

**Table 1:** Sequences of primer sets for amplification of *OvNad* subunits.

Primer	Sequences (from 5′ to 3′)
*OvNad*1 (F)	GATTACGCAGAAGCGGAGAG
*OvNad*2 (F)	TAGCTCAAGGGCTTCTTTGG
*OvNad*4 (F)	GGCTTCCAATGTTGCTCTGT
*OvNad*5 (F)	TTTGCGGAGGTTTGTTACCT
*OvNad*1 (R)	AAGAGTAGCACGAGCCCAGA
*OvNad*2 (R)	ACTGCTACTGAACCCGGAAA
*OvNad*4 (R)	TACCGAAACAGGCCTACTGG
*OvNad*5 (R)	CACCTCACCAATTCAACACG

**Table 2:** The number of parasite-positive samples by PECT and PCR amplification of *OvNad* subunits.

Parasites	Positive samples by PECT	Positive samples by PCR amplification of			
		*OvNad1*	*OvNad2*	*OvNad4*	*OvNad5*
*O*. *viverrini*	75	48	66	60	75
Hookworm	11	0	0	0	0
*A*. *lumbricoides*	3	0	0	0	0
*Taenia* spp.	3	0	0	0	0
*S*. *stercoralis*	5	0	0	0	0
*T*. *trichiura*	6	0	0	0	0
Hookworm + *A*. *lumbricoides*	5	0	0	0	0
Hookworm + *T*. *trichiura*	1	0	0	0	0
*A*. *lumbricoides* *+* *E*. *vermicularis*	2	0	0	0	0
*A*. *lumbricoides* *+* *T*. *trichiura*	1	0	0	0	0
*Taenia* spp + *E*. *vermicularis*	2	0	0	0	0
No parasite detection	52	0	0	0	2
Total	166	48	66	60	77

**Table 3:** Sensitivity, specificity, positive predicted value (PPV), and negative predicted value (NPV) of OvNad subunit detection by PCR amplification compared with PECT.

*OvNad* subunits	Sensitivity (%)	Specificity (%)	Positive predicted value (PPV)	Negative predicted value (NVP)
*OvNad*1	64.00	100.00	100.00	77.12
*OvNad*2	88.00	100.00	100.00	91.00
*OvNad*4	80.00	100.00	100.00	85.85
*OvNad*5	100.00	100.00	100.00	100.00

## Data Availability

All data used to support the findings of this study are available from the corresponding author upon request.
